# Somatic cell selection for chlorsulfuron-resistant mutants in potato: identification of point mutations in the acetohydroxyacid synthase gene

**DOI:** 10.1186/s12896-017-0371-4

**Published:** 2017-06-06

**Authors:** Philippa J. Barrell, Julie M. Latimer, Samantha J. Baldwin, Michelle L. Thompson, Jeanne M.E. Jacobs, Anthony J. Conner

**Affiliations:** 1 The New Zealand Institute for Plant & Food Research Limited, Private Bag 4704, Christchurch, 8140 New Zealand; 2 Bio-Protection Research Centre, Lincoln University, PO Box 85084, Lincoln, 7647 New Zealand; 3 AgResearch Ltd, Lincoln Research Centre, Private Bag 4749, Christchurch, 8140 New Zealand

**Keywords:** Acetohydroxyacid synthase, Acetolactate synthase, Chlorsulfuron resistance, Intragenic selectable marker, Potato, Somatic cell selection, Sulfonylurea resistance

## Abstract

**Background:**

Somatic cell selection in plants allows the recovery of spontaneous mutants from cell cultures. When coupled with the regeneration of plants it allows an effective approach for the recovery of novel traits in plants. This study undertook somatic cell selection in the potato (*Solanum tuberosum* L.) cultivar ‘Iwa’ using the sulfonylurea herbicide, chlorsulfuron, as a positive selection agent.

**Results:**

Following 5 days’ exposure of potato cell suspension cultures to 20 μg/l chlorsulfuron, rescue selection recovered rare potato cell colonies at a frequency of approximately one event in 2.7 × 10^5^ of plated cells. Plants that were regenerated from these cell colonies retained resistance to chlorsulfuron and two variants were confirmed to have different independent point mutations in the *acetohydroxyacid synthase* (*AHAS*) gene. One point mutation involved a transition of cytosine for thymine, which substituted the equivalent of Pro-197 to Ser-197 in the AHAS enzyme. The second point mutation involved a transversion of thymine to adenine, changing the equivalent of Trp-574 to Arg-574. The two independent point mutations recovered were assembled into a chimeric gene and binary vector for *Agrobacterium*-mediated transformation of wild-type ‘Iwa’ potato. This confirmed that the mutations in the *AHAS* gene conferred chlorsulfuron resistance in the resulting transgenic plants.

**Conclusions:**

Somatic cell selection in potato using the sulfonylurea herbicide, chlorsulfuron, recovered resistant variants attributed to mutational events in the *AHAS* gene. The mutant *AHAS* genes recovered are therefore good candidates as selectable marker genes for intragenic transformation of potato.

**Electronic supplementary material:**

The online version of this article (doi:10.1186/s12896-017-0371-4) contains supplementary material, which is available to authorized users.

## Background

Somatic cell selection in plants applies the principles of microbial genetics to plant cell cultures. In this manner a selection pressure is imposed on a large population of cultured cells so that only rare individuals with a specific phenotype are capable of survival or growth [1]. Effective use of this technology allows the recovery of spontaneous mutants from cell cultures with the potential recovery of novel traits with agricultural applications in crop plants. The high aptitude of potato (*Solanum tuberosum* L.) for performance in cell culture and the ease of shoot regeneration, offers the opportunity for employing this approach for somatic improvement [2].

Despite this potential, somatic cell selection has generated only a few examples of novel traits in potato. These include cell lines with resistance to culture filtrates of *Fusarium oxysporum* [3] and high salt concentration [4]. The most important application of somatic cell selection in potato improvement involves the development of clones with resistance to common scab disease incited by *Streptomyces scabiei* [5, 6]. The use of thaxtomin A (the pathotoxin of *S. scabiei*) as a selection agent on cultured cells allowed the recovery of rare resistant cell lines that could be regenerated into plants with improved resistance to common scab disease following both greenhouse and field evaluation [6, 7].

Herbicide resistance is another important trait in crop plants with applications for weed control, crop management and seed production [8]. Herbicides such as the sulfonylureas and imidazolinones target the acetohydroxyacid synthase enzyme (AHAS) [also known as acetolactate synthase (ALS)], thereby preventing the biosynthesis of branched chain amino acids, resulting in plant death [9]. Chemical mutagenesis and subsequent breeding have yielded plants with sulfonylurea and imidazolinone resistance in wheat (*Triticum aestivum*), barley (*Hordeum vulgare*), sugarcane (*Saccharum* spp.) and rice (*Oryza sativa*) [10–13], as well as forage brassicas including swede (*B. napus*), rape (*B. napus*) and turnips (*B. rapa*) [14]. Furthermore, biotypes of 159 plant species have evolved resistance to AHAS-inhibitors globally [15]. In most cases resistance to AHAS-inhibitor herbicides is due to point mutations in the coding region of the *AHAS* gene [9, 16]. *AHAS* genes with these point mutations have been shown to confer resistance to sulfonylurea- and imidazolinone-based herbicides when transformed into plants [17–21]. Somatic cell selection has also been used to select for resistance to sulfonylurea herbicides in tobacco (*Nicotiana tabacum*) [22] and sugarbeet (*Beta vulgaris*) [23, 24].

This study provides another example of successfully producing a novel trait in potato (resistance to the sulfonylurea herbicide, chlorsulfuron) using somatic cell selection. Rare cell colonies were recovered following exposure of cultured potato cells to a concentration of the sulfonylurea herbicide, chlorsulfuron, which normally inhibits cell growth. Plants regenerated from these cell colonies retained resistance to chlorsulfuron and two variants were confirmed to have different point mutations in the *AHAS* gene. The two independent point mutations recovered were assembled into a chimeric gene and binary vector for *Agrobacterium*-mediated transformation. Wild-type potato cultivar ‘Iwa’ was transformed with this vector and the resulting plants were confirmed to exhibit chlorsulfuron resistance. This validates the point mutations identified in the potato *AHAS* gene to be the source of resistance to chlorsulfuron and provides a potato-derived selectable marker gene to facilitate intragenic/cisgenic gene transfer to potato [25, 26].

## Methods

### Plant material

Micropropagated virus-free plants of potato cultivar ‘Iwa’ were maintained as previously described [27] on plant multiplication (PM) medium consisting of MS salts and vitamins [28] plus 30 g/l sucrose, 40 mg/l ascorbic acid and 500 mg/l casein hydrolysate. After the pH was adjusted to 5.8 with 0.1 M KOH, 8 g/l agar was added and the medium was autoclaved at 121 °C for 15 min. Aliquots of 50 ml were dispensed into pre-sterilized plastic containers (80 mm diameter × 50 mm high; Vertex Plastics, Hamilton, New Zealand). Plants were routinely subcultured as two- to three-node segments every three–four weeks and grown at 26 °C under cool white fluorescent lamps (80–100 μmol/m^2^/s; 16-h photoperiod). To define culture conditions and chlorsulfuron concentrations for effective cell selection, initial experiments were conducted on micropropagated potato plants in PM medium with and without casein hydrolysate and supplemented with 0 to 20 μg/l chlorsulfuron (filter sterilized). In some experiments 100 mM each of leucine, isoleucine and valine (filter sterilized) was added to the culture medium.

### Cell culture and selection

Three weeks after the previous subculture, callus cultures were initiated from stem internodes (*ca.* 1 cm long) cut in half lengthways and placed face down on potato callus induction (CI) medium, composed of the PM medium supplemented with 2 mg/l BAP and 0.2 mg/l NAA and dispensed as 25 ml aliquots into standard plastic Petri dishes (9 cm diameter × 1 cm high). After four weeks’ incubation in darkness at 22 °C, stems showing good callus production were transferred to 40 ml of liquid CI medium in 100 ml Erlenmeyer flasks and incubated on an orbital shaker (110 rpm) for 5 days. To remove stem material and clumps of callus tissue, the resulting cell culture was poured through a stainless steel sieve (0.5 mm pores) mounted over a series of 50 mL Falcon™ tubes and left standing until a clear line of separation was visible to determine the cell suspension volume. The supernatant was discarded and the settled cells resuspended in four volumes of fresh CI medium without casein hydrolysate. The cycle of cell settling and resuspension was then repeated, with the culture medium supplemented with 20 μg/l chlorsulfuron (filter sterilized). The cell suspension was incubated for 5 days on an orbital shaker as described above.

### Variant recovery and plant regeneration

The chlorsulfuron-treated cells were allowed to settle in 50 ml Falcon tubes, cell density was adjusted to about 10^5^ plating units per ml, and 0.5 ml was plated onto CI medium in standard Petri dishes for the recovery of surviving potato cells. To assist the rescue of putative rare chlorsulfuron-resistant variant potato cells, a nurse culture of *Nicotiana plumbaginifolia* cells, cultured as previously described [29], was established by plating 0.8 ml of a cell suspension culture (10^5^ plating units per ml) directly onto a fresh plate of CI medium. A piece of sterile 7 cm diameter Whatman No. 1 filter paper was placed over the plated *N. plumbaginifolia* cells, with the chlorsulfuron-treated potato cells being plated on top of the filter paper. The plated cells were grown at 26 °C under cool white fluorescent lamps (30–40 μmol/m^2^/s; 16-h photoperiod). After two–three months, rescued potato cell colonies were transferred to callus regeneration medium (PM medium with sucrose reduced to 5 g/l sucrose, plus 1 mg/l zeatin and 5 mg/l GA_3,_ both filter sterilized) and incubated under reduced light intensity (10 μmol/m^2^/s; 16-h photoperiod) at 26 °C for a further two–three months with calli being transferred to fresh media every four–five weeks. The first regenerated shoot from each variant was transferred to PM medium for micropropagation and subsequent screening for chlorsulfuron resistance.

### PCR isolation of the *AHAS* gene from wild-type potato cultivar ‘Iwa’

Genomic DNA was isolated from *in vitro* shoots of the potato cultivar ‘Iwa’ and the variants CR06 and CR27 derived from ‘Iwa’, based on the method previously described [30].

The primers AHAS1F (5’ TAGCCATTTTGCCTCCTTTC 3’) and AHAS1R (5’ CAACGGCAAACTAGACAGATAGAA 3’) were used to amplify wild-type potato cultivar ‘Iwa’ *AHAS* coding sequences via PCR. Reactions consisted of 1x PWO buffer and 2.5U PWO (Roche Diagnostics N.Z., Ltd. Auckland New Zealand), 20 mM MgSO_4_, 0.2 mM each dNTP, 0.1 μM AHAS1F, 0.1 μM AHAS1R, and 50 ng Iwa DNA. PCR conditions included an initial denaturing step of 94 °C for 2 min, 10 cycles of 94 °C for 15 s, 59 °C for 30s, 72 °C for 2 min. A further 20 cycles of 94 °C for 15 s, 59 °C for 30s and 72 °C for 2 min followed with an extension of 5 s per cycle and a 7 min 72 °C final extension step. Amplified products were separated by electrophoresis in a 1% agarose gel in 1xTAE buffer and visualized under UV light after staining with ethidium bromide.

### Cloning and sequencing of PCR products

PCR fragments of approximately 2 kb were extracted from agarose gel using a QIAquick gel extraction kit (QIAGEN, Hilden, Germany) and ligated into the pGEM®**-**T Easy vector (Promega, Mannheim, Germany). The ligation reactions were transformed into Subcloning Efficiency^TM^ DH5α^TM^ Competent Cells (Invitrogen, Carlsbad, CA, USA) according to manufacturer’s instructions. Transformations were plated on LB media supplemented with 100 mg/l ampicillin, 100 mM/l isopropyl β-D-1-thiogalactopyranoside (IPTG) and 20 mg/l X-gal. Plasmid DNA from 16 white colonies from wild-type ‘Iwa’ were isolated using High Pure Plasmid Isolation Kit (Roche Applied Science) and sequenced using Applied Biosystems BigDye® Terminator v3.1 kit. Initial sequencing reactions were performed with M13 forward and reverse primers. Further sequencing primers were designed within the *AHAS* sequence as follows (5’-3’) ALSWALKER1: TGTACGCCAAATCAAAAA; AHASF: GCCTCACCATCTCCATGTTT; Alsprom: CGATGATGATGGGTGTGGGTGAGA; ALS5#2: CCTCGGCACTTGACGGCTAA; ALS5#3: AAAACGCTTCACGAACAACC; als3#3: GAAGCCATCCCTCCACAATA; ALS3#2: TTAGGAGCAATGGGATTTGG; ALSterm: GGGCCATACTTGTTGGATGT; AHAS1R: CAACGGCAAACTAGACAGATAGAA; AHASR: CCGTCTTATGCCAACCATTT. Sequencing reactions were analyzed using an ABI 3130xl automated sequencer (Applied Biosystems, Foster City, USA).

### Binary vector construction and plant transformation

A chimeric *AHAS* gene was designed to incorporate the two point mutations in the *AHAS* coding region uncovered by the somatic cell selection experiments. The modified *AHAS* coding region was designed to be flanked by the promoter and terminator regions of the *Lhca3* gene from potato cultivar ‘Iwa’ that we have previously described [31]. The *AHAS* coding sequence flanked by the *Lhca3* promoter and terminator was obtained from Genscript Corporation (Piscataway, NJ, USA) as a fragment ligated into the plasmid pUC57. The chimeric *AHAS* gene was removed from the pUC57 backbone by digestion with the enzyme *Mlu*I. The fragment was blunt-ended and ligated into the binary vector pMOA33 [32], previously digested with the blunt cutting enzyme *Pme*I. Ligation reactions were transformed into *E. coli* strain DH5α. Plasmid DNA isolated from transformed *E. coli* colonies was digested with the restriction enzyme *Eco*RI to identify the orientation of the inserted fragment. A binary vector with the *nptII* selectable marker gene and the chimeric *AHAS* gene in the same orientation was identified and named pMOA33AHAS. The binary vector was transferred to the disarmed *Agrobacterium tumefaciens* strain EHA105 [33]. *Agrobacterium*-mediated transformation of virus-free potato cultivar ‘Iwa’ plantlets was performed following our standard method [34]. Kanamycin (100 mg/l) was used as the selection agent, and regenerated plants were challenged with media containing 20 μg/l chlorsulfuron to assess their ability for roots to grow into the media containing chlorsulfuron.

### Molecular assessment of regenerated plants

Genomic plant DNA was extracted using a Macherey-Nagel NucleoSpin® Plant II Kit. (Düren, Germany). The primers (5’-3’) pMOA33RBFor: CCCAGTAGCTGACATTCATC; and StCabAHASSeqRev: CCCCTCCCCTTCTCTTATGTGTA were used in PCRs to confirm transgenic status with an expected 370 bp amplicon spanning the *Lhca*3 terminator of the construct into the *nos* promoter of the selectable marker from the binary vector pMOA33 [32]. The same DNA samples were also assessed via PCR for the presence of *Agrobacterium* using primers based on the *virG* gene producing a predicted amplicon of 692 bp [31].

## Results

### Recovery of chlorsulfuron-resistant potato plants

Initial experiments investigated the influence of culture medium composition and chlorsulfuron concentrations on *in vitro* potato plants (Additional file 1: Figure S1). The presence of up to 20 μg/l chlorsulfuron in the standard PM medium containing casein hydrolysate inhibited, but did not prevent, root development on *in vitro* potato plants. However, upon omission of casein hydrolysate, the toxicity of chlorsulfuron was substantially greater with no root formation observed in medium with 20 μg/l chlorsulfuron. Supplementing the latter medium with branched chain amino acids (leucine, isoleucine and valine) mitigated the toxic effects of chlorsulfuron.

A dose response experiment using PM medium without casein hydrolysate determined that 20 μg/l chlorsulfuron was the lowest concentration that prevented root development on all plants (Additional file 2: Figure S2). Similar experiments were repeated with leaf and stem explants placed on CI medium with and without casein hydrolysate. This confirmed that in the absence of casein hydrolysate, 20 μg/l chlorsulfuron completely inhibited callus induction from these explants, whereas only a very slight callus growth was observed at 10 μg/l chlorsulfuron. As expected, the inclusion of branched chain amino acids (leucine, isoleucine and valine) in the medium substantially reduced the toxic effects of chlorsulfuron, whereas the presence of casein hydrolysate partially alleviated the toxicity.

**Fig. 1:**
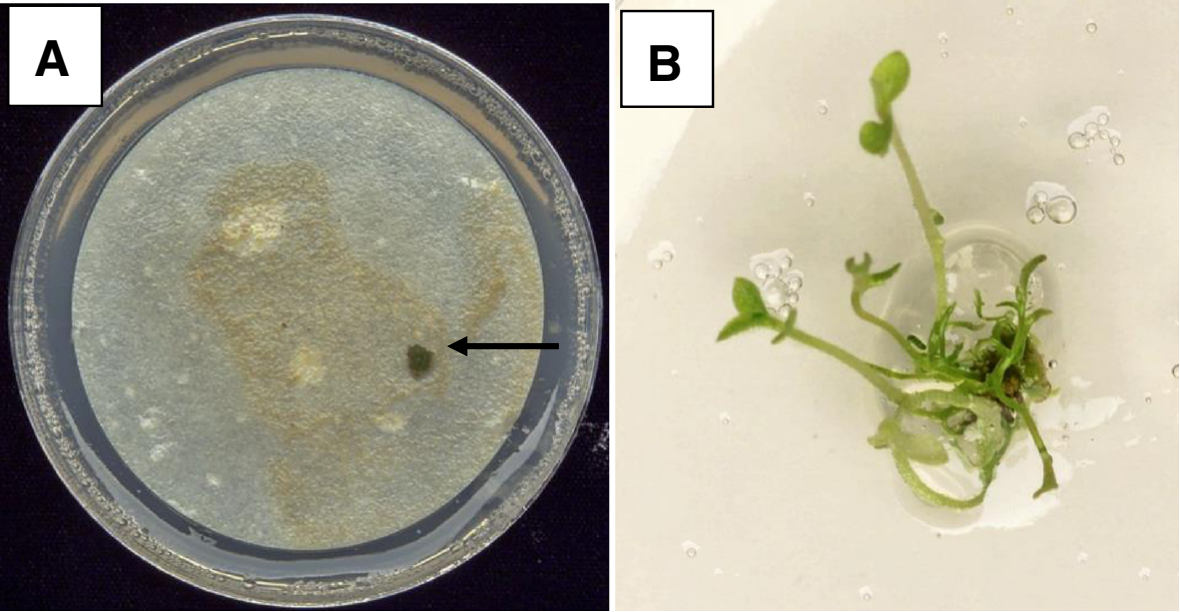
Somatic cell selection and regeneration of chlorsulfuron resistance in potato. **a** The *arrow* indicates a rare potato cell colony rescued from cell suspension culture growing on media containing 20 μg/l chlorsulfuron (each Petri dish was seeded with approximately 5 × 10^4^ cells) **b** An *in vitro* potato plant regenerated from a cell colony with resistance to chlorsulfuron

**Fig. 2:**
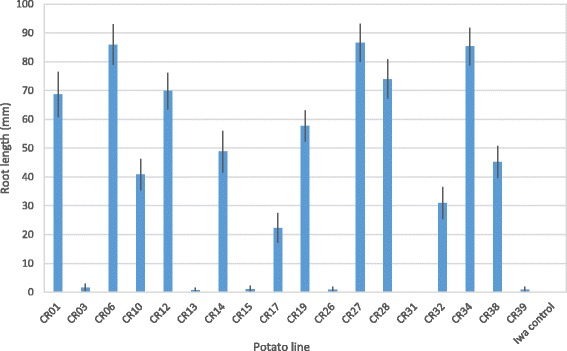
Root length of plants from regenerated cell colonies. Length of longest root on micropropagated plants (mean ± standard deviation; *n* = 10) after 3 weeks cultured on PM medium (without casein hydrolysate) plus 20 μg/l chlorsulfuron. The twelve resistant variants were: CR01, CR06, CR10, CR12, CR14, CR17, CR19, CR27, CR28, CR32, CR34, and CR38. ‘Iwa’ control plants did not form roots into the media

The exposure of potato cells to 20 μg/l chlorsulfuron in suspension culture for five days prior to plating on CI medium resulted in the complete inhibition of the background growth of wild-type cells during the first month of incubation. After two to three months of incubation, rare variant cell colonies were observed (Figure 1A). A total of 42 variant cell colonies were recovered from 64 Petri dishes, of which 22 retained chlorsulfuron resistance upon a further subculture cycle (Table 1). Eighteen of these variants with stable resistance to chlorsulfuron as callus cultures were successfully regenerated into plants (Fig. 1b), of which 12 continued to exhibit resistance to chlorsulfuron as whole plants (Table 1, Fig. 2). The variants CR06, CR27 and CR34 exhibited the highest degree of resistance to chlorsulfuron as judged by developing the longest roots on culture medium supplemented with 20 μg/l chlorsulfuron (Fig. 2). The variant CR34 displayed atypical shoot morphology with thin spindly stems and small curved leaves and was not investigated further.

**Table 1:** Summary of variants rescued following exposure of somatic cells of potato to chlorsulfuron

Number of selection plates^a^	64
Number of variant colonies initially recovered	42
Number of variants retaining chlorsulfuron resistance	22
Number of variants regenerating plants	18
Number of variants with chlorsulfuron-resistant plants^b^	12

^a^Each plate was seeded with 5 × 10^4^ potato cells previously exposed to 20 μg/l chlorsulfuron

^b^See Fig. 2

### *AHAS* sequence results from potato cultivar ‘Iwa’ and the chlorsulfuron-resistant plants

DNA sequencing of the *AHAS* gene amplified from potato cultivar ‘Iwa’ revealed a 1980 bp gene composed of a single exon with no introns and encoding a predicted protein of 659 amino acids. The sequencing revealed three distinct alleles in ‘Iwa’, with the sequence of one allele deposited in GenBank (Accession HM114275).

Interrogation of version 4.03 of the potato genome database [35, 36] revealed three matches of accession HM114275 to the diploid reference potato genome sequence of DM1-3 516 R44 (‘DM’), on chromosomes 3, 6 and 7 (Additional file 3: Figure S3). The locus on DM chromosome 6 showed only partial alignment with bases 985–2028 of accession HM114275. Our sequence derived from ‘Iwa’ (Accession HM114275) showed 99.5% identity to the locus on DM chromosome 3, with only 95.4% identity to the locus on chromosome 7.

**Fig. 3:**
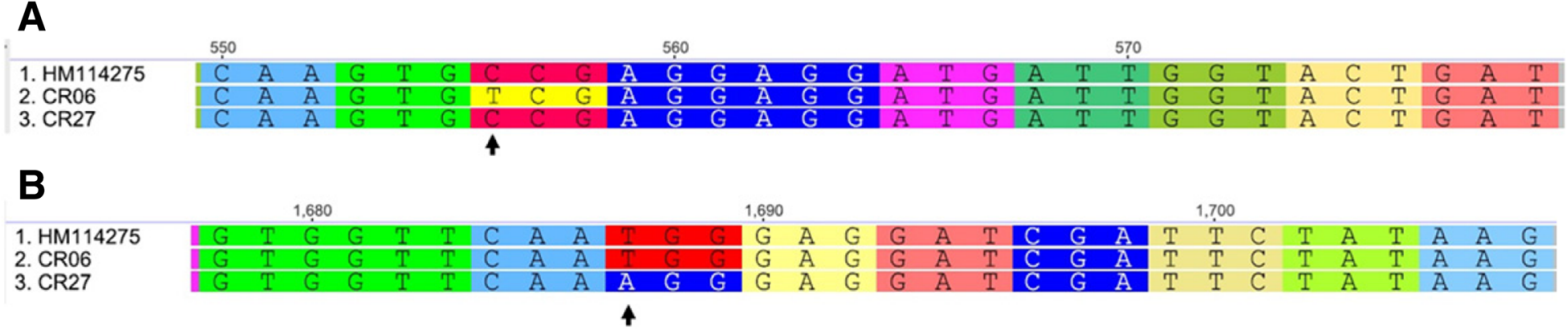
The nucleotide changes responsible for conferring resistance to chlorsulfuron in potato variants CR06 and CR27. Numbering of nucleotides is according to the potato cultivar ‘Iwa’ sequence (GenBank accession HM14275) where the A of the starting methionine residue is 1. *Arrows* indicate point mutations conferring resistance to chlorsulfuron. Codons are highlighted according to their translation, and where the point mutations cause an amino acid change, the highlighted colour is changed. **a** *AHAS* nucleotides 550–579: Line 1: ‘Iwa’; Line 2: Potato variant CR06; Line 3: Potato variant CR27. **b** *AHAS* nucleotides 1678–1707. Line 1: ‘Iwa’; Line 2: Potato variant CR06; Line 3: Potato variant CR27

PCR amplification and DNA sequencing of the *AHAS* coding region from plants regenerated from cell colonies CR06 and CR27 uncovered two separate point mutations (Fig. 3). In CR06, a transition mutation occurred at position 556 with the substitution of cytosine for thymine, which changes the 186th amino acid from a proline into a serine residue. Whereas in CR27 a transversion mutation occurred at position 1687 with the substitution of thymine with adenine, changing the 563rd amino acid from tryptophan to arginine.

### Potato transformation with an *AHAS* sequence containing the two point mutations

The coding region of the potato *AHAS* gene used for transformation was designed to encompass the two point mutations identified from CR06 and CR27 (Additional file 4: Figure S4). This sequence with the regulatory controls of the potato *Lhca3* gene was synthesized and the resulting chimeric gene inserted into a binary vector to produce pMOA33AHAS (Additional file 5: Figure S5). *Agrobacterium*-mediated transformation of potato with pMOA33AHAS using kanamycin as the selective agent yielded 15 independently regenerated kanamycin-resistant plants. Potato transformation using the same vector with chlorsulfuron as the selective agent failed to produce chlorsulfuron-resistant plants.

**Fig. 4:**
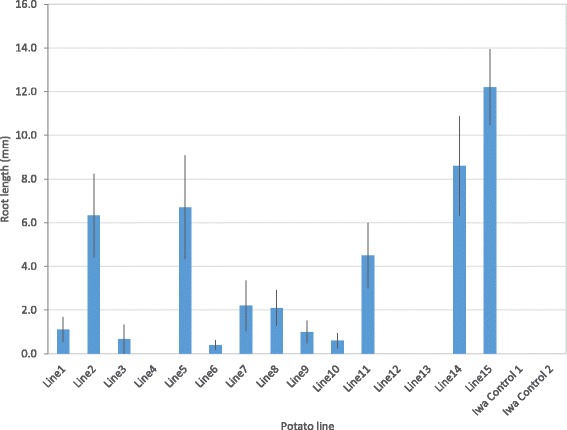
Root lengths of plants transformed with the binary vector pMOA33AHAS cultured on PM medium (without casein hydrolysate) plus 20 μg/l chlorsulfuron. The length of longest root (mm) after six weeks is plotted (mean ± standard error; *n* = 10 except lines 1–4 where *n* = 9)

All 15 kanamycin resistant-plants were confirmed to contain the chimeric *AHAS* gene by PCR with the amplification of a 370 bp fragment (data not shown). The PCR reactions performed using primers to amplify *virG* from *Agrobacterium* were all negative and showed that there was no contaminating *Agrobacterium* DNA in the plant DNA samples (data not shown). The majority of the transgenic lines (12 of 15) exhibited root growth on *in vitro* plants when challenged on media containing 20 ug/ml chlorsulfuron, with five lines (#2, 5, 11, 14, and 15) exhibiting substantial root growth (Fig. 4).

## Discussion

This study reports the development of a somatic cell selection system using chlorsulfuron as a positive selection agent to generate herbicide resistance in potato plants. The approach used involved a rescue selection strategy with cell suspension cultures being exposed to the selection agent for five days prior to plating onto a medium without selection for the recovery of surviving variant cell colonies (Fig. 1a). The advantages of a rescue selection approach are the uniform exposure of cells to the selection agent and the rapid recovery of resistant variants on a standard medium [1].

Effective somatic cell selection in plants depends on defining the lowest concentration of the selection agent that completely prevents the growth of all wild-type cells. A key component for establishing an effective somatic cell selection approach for chlorsulfuron resistance in potato involved understanding the interaction between the culture medium and the selection agent. Cell culture media for many plant species often include complex ingredients; in the case of potato, a common ingredient is casein hydrolysate (e.g. [17, 27]), a complex mixture that includes amino acids. Chlorsulfuron was more toxic to potato cultures in the absence of casein hydrolysate or branched chain amino acids (Additional file 1: Figure S1). The alleviation of chlorsulfuron toxicity by supplementing the culture medium with branched chain amino acids is well documented [37]. This is expected given that the target site for chlorsulfuron and related herbicides is acetohydroxyacid synthase, the first enzyme in the pathway for biosynthesis of branched chain amino acids [9]. The omission of casein hydrolysate from the culture medium is therefore essential during the selection and subsequent characterization of variants for chlorsulfuron resistance in potato. This is consistent with the need to omit casein hydrolysate from the culture medium when using the *bar* gene as a selectable marker for potato transformation with phosphinothricin as the selection agent [38].

Following rescue selection, assessment of variant stability and plant regeneration 12 variants continued to exhibit resistance to chlorsulfuron as whole plants (Table 1, Fig. 2). These 12 variant plants with chlorsulfuron resistance were recovered from a total of 64 selection plates each seeded with 50,000 cells. This represents approximately one event in 2.7 × 10^5^ somatic cells plated. This frequency is consistent with effective somatic cell selection in plants [1], and very similar to the rescue selection approach used for thaxtomin A resistance in potato [5, 6].

The highest degree of chlorsulfuron resistance was observed in potato variants CR06, CR27 and CR34 (Fig. 2). The plants of one of these variants (CR34) grew poorly and exhibited unusual shoot morphology, presumably as a consequence of undesirable somaclonal changes during the cell culture phase of somatic cell selection. Such events are common during plant tissue culture [1] and are frequently reported in regenerated potato clones in addition to desired genetic change [34]. Further analysis of the potato variants therefore focused on CR06 and CR27 in order to establish a mutational basis for the phenotypic change of higher chlorsulfuron resistance. Resistance to sulfonylurea herbicides, such as chlorsulfuron, is usually associated with point mutations in the *AHAS* gene resulting in amino acid substitutions that prevent or reduce herbicide binding to the AHAS enzyme [9]. We therefore sequenced the *AHAS* gene from the potato cultivar ‘Iwa’, then repeated this for the CR06 and CR27 variants derived from ‘Iwa’.

The sequence of the coding region of the *AHAS* gene from ‘Iwa’ (GenBank accession HM114275) showed the closest match to a locus on potato chromosome 3 (Additional file 1: Figure S1). At this locus the sequencing revealed three distinct alleles in ‘Iwa’, with one of the alleles presumably in the duplex state for an autotetraploid potato cultivar. Sequencing the *AHAS* coding region from variant plants designated CR06 and CR27 revealed that each possessed different independent point mutations of significance for explaining the resistance to AHAS-inhibitors (Fig. 3). This confirms that CR06 and CR27 are derived from mutation events with at least one of the four alleles in tetraploid potato conferring herbicide resistance in the expected dominant condition.

For AHAS it is common practice to standardize amino acid number to the *Arabidopsis thaliana* sequence [9, 16]. Pro-186 and Trp-563 in potato correspond to Pro-197 and Trp-574 in *A. thaliana*. The vast majority of species with resistance to AHAS-inhibitors have amino acid substitutions at either of these two sites [9, 16]. The Pro-197 to Ser-197 mutations, as found in CR06 potato in this study, is very common across many species [16]. This mutation is known to prevent chlorsulfuron binding to AHAS and to have no effect on AHAS enzyme kinetics and functionality [39]. At Trp-574, a substitution to Leu-574 is the most frequent recorded mutation for resistance to AHAS-inhibitors across many species [16], although many other amino acid substitutions have been recorded in this position, including Arg-574 as found in CR27 in this study [9].

The point mutations revealed in variants CR06 and CR27 result in amino acid substitutions that are well known to confer resistance to AHAS-inhibitor herbicides [9, 16]. Introduction of a gene with these mutations back into wild-type potato cultivar ‘Iwa’ using *Agrobacterium*-mediated transformation with a binary vector confirmed that the mutations in the *AHAS* gene conferred chlorsulfuron resistance in the resulting transgenic plants (Fig. 4). This therefore establishes that the variants recovered via somatic cell selection in potato can be attributed to mutational events.

Similar to the results on potato in this study, somatic cell selection for chlorsulfuron resistance in tobacco [22] was determined to result from mutations causing amino acid substitutions equivalent to Pro-197 and Trp-574 in the *AHAS* gene [40]. Mutations at both sites were capable of independently conferring resistance to chlorsulfuron, whereas the combination of both amino acid changes led to a higher level of resistance to chlorsulfuron [40]. Therefore, we decided to combine the two point mutations identified in our potato somatic cell selection experiments into a single construct for our transformation experiments. A synthetic DNA sequence was assembled combining both point mutations recovered from our somatic cell selection experiments into the *AHAS* coding region (Additional file 4: Figure S4) flanked by the promoter and 3’ terminator regions of the potato *Lhca3* gene. This chimeric intragene was ligated into the binary vector pMOA33 which has a *nptII* selection marker gene conferring kanamycin resistance [32]. The resulting pMOA33AHAS was used in *Agrobacterium*-mediated transformation with both kanamycin and chlorsulfuron as selective agents in separate transformation experiments with ‘Iwa’. Transgenic plants were recovered as expected using kanamycin as a selection agent. However, we did not recover transgenic plants using chlorsulfuron as a selective agent with pMOA33AHAS. This may be a consequence of the mutant *AHAS* gene not expressing well in cell and tissue culture due to the *Lhca*3 promoter which has light- and leaf-specific expression [41, 42].

The transgenic plants obtained using kanamycin as a selection agent were resistant to chlorsulfuron, as judged by the growth of roots into media containing chlorsulfuron compared with no roots on the wild-type control plants (Fig. 4). We observed that the root length of transgenic plants containing the pMOA33AHAS T-DNA that were challenged on media containing chlorsulfuron was substantially shorter than the variant plants isolated from the cell selection experiments (Figs. 2 and 4). We hypothesize that the differences may be due to the promoters driving the mutant *AHAS* gene in each group of potato plants. Interrogation of the supplementary data published with the potato genome sequence [35] reveals that members of the *Lhca*3 gene family are at much reduced expression compared with the *AHAS* genes in tuber, stolon and root tissues (data not shown). As the potato *Lhca*3 gene is light-induced and leaf-specific [41, 42], the reduced root length for the transgenic lines compared with the variant lines following cell selection is expected. The marked variation in root length among the independently derived transgenic lines, with five transgenic lines having substantially higher chlorsulfuron resistance (Fig. 4), is probably a consequence of transgene position effects following plant transformation [43, 44] rather than expression driven by the *Lhca*3 promoter.

*Agrobacterium*-mediated plant transformation traditionally involves the transfer of bacterial and other non-plant DNA into plants [26]. One of the concerns of the general public about this technology involves the transfer of genetic material between species that cannot normally hybridize [25, 45, 46]. Intragenic and cisgenic technologies have been developed to address the concerns of “crossing the species barrier” in the construction of transformed plants. Both intragenic and cisgenic strategies involve the transfer of DNA derived from the species own gene pool [25, 26, 45, 47, 48]. One of the limiting steps for developing intragenic and cisgenic plants is the availability of selectable marker genes for transformation [25, 26]. The mutant *AHAS* genes recovered in this study are good candidates as a selectable marker gene for intragenic transformation of potato.

## Conclusion

The sulfonylurea herbicide, chlorsulfuron, was used as a positive selection agent for somatic cell selection in the potato cultivar ‘Iwa’. This recovered rare potato cell colonies at a frequency of approximately one event in 2.7 × 10^5^ of plated cells and plants regenerated from these cell colonies retained resistance to chlorsulfuron. Two variants were found to have different independent point mutations in the *AHAS* gene. *Agrobacterium*-mediated transformation of wild-type ‘Iwa’ potato with a chimeric gene containing the two mutations confirmed that they conferred chlorsulfuron resistance. The mutant *AHAS* genes provide candidates as selectable marker genes for intragenic transformation of potato.

## Additional files

Additional file 1: Figure S1. Influence of culture medium composition and chlorsulfuron concentrations on *in vitro* plants of wild-type potato cultivar ‘Iwa’. Root length was measured after three weeks with mean root length (mm) ± standard deviation plotted (*n* = 30). PM 0 = potato multiplication media, no chlorsulfuron; PM 10 = potato multiplication media, 10 μg/l chlorsulfuron; PM 20 = potato multiplication media, 20 μg/l chlorsulfuron; PM –ch 0 = potato multiplication media, no casein hydrolysate, no chlorsulfuron; PM –ch 10 = potato multiplication media, no casein hydrolysate, 10 μg/l chlorsulfuron; PM –ch 20 = potato multiplication media, no casein hydrolysate, 20 μg/l chlorsulfuron; PM-ch + aa 0 = potato multiplication media, no casein hydrolysate, amino acids, no chlorsulfuron; PM-ch + aa 10: potato multiplication media, no casein hydrolysate, amino acids, 10 μg/l chlorsulfuron; PM-ch + aa 20 = potato multiplication media, no casein hydrolysate, amino acids, 20 μg/l chlorsulfuron; amino acids refers to the presence of 100 mM each of leucine, isoleucine and valine. (DOCX 16 kb)

Additional file 2: Figure S2. A dose response experiment of chlorsulfuron on growth of wild-type potato cultivar ‘Iwa’ using PM medium without casein hydrolysate. Root length was measured after three weeks with mean root length (mm) ± standard deviation plotted (*n* = 30). (DOCX 15 kb)

Additional file 3: Figure S3. Alignment of the coding sequence of the wild-type potato cultivar ‘Iwa’ *AHAS* allele (GenBank accession HM114275) and partial DNA BAC sequences from the reference potato genome [35, 36]. Line 1: PGSC0003DMB000000227, which maps to chromosome 6; Line 2: PGSC0003DMB000000368, which maps to chromosome 3; Line 3: PGSC0003DMB000000096 which maps to chromosome 7; and Line 4: The coding region of the AHAS allele from ‘Iwa’, GenBank accession HM114275. (DOCX 1863 kb)

Additional file 4: Figure S4. Nucleotide sequence of the mutated potato *AHAS* coding region. The two nucleotides highlighted in red are the point mutations identified in the somatic cell selection experiments from cell colonies CR06, CR27. The first mutation, the highlighted residue at nucleotide 556 was originally C and was converted to a T, which changes the 186th amino acid from a proline into a serine residue. The second highlighted residue was identified to have a mutation at position 1687, where a T residue was converted to A, which changed the 563rd amino acid from tryptophan into an arginine residue. (DOCX 12 kb)

Additional file 5: Figure S5. The binary vector pMOA33-AHAS. The locations of the primers pMOA33RBFor and StCabAHASSeqRev used to confirm transgenic status of the regenerated plants are indicated. (DOCX 68 kb)

## Abbreviations

AHAS: acetohydroxyacid synthase
ALS: Acetolactate synthase
CI: Callus induction
PCR: Polymerase chain reaction
PM: Plant multiplication
