# Supplementary material for: Somatic cell selection for chlorsulfuron-resistant mutants in potato: identification of point mutations in the acetohydroxyacid synthase gene
Source: BMC Biotechnol. 2017 Jun 6;17:49. doi: 10.1186/s12896-017-0371-4 (PMC5461709; doi:10.1186/s12896-017-0371-4)
Supplement: Supplementary file 1 — Influence of culture medium composition and chlorsulfuron concentrations on in vitro plants of wild-type potato cultivar ‘Iwa’. Root length was measured after three weeks with mean root length (mm) ± standard deviation plotted (n = 30). PM 0 = potato multiplication media, no chlorsulfuron; PM 10 = potato multiplication media, 10 μg/l chlorsulfuron; PM 20 = potato multiplication media, 20 μg/l chlorsulfuron; PM –ch 0 = potato multiplication media, no casein hydrolysate, no chlorsulfuron; PM –ch 10 = potato multiplication media, no casein hydrolysate, 10 μg/l chlorsulfuron; PM –ch 20 = potato multiplication media, no casein hydrolysate, 20 μg/l chlorsulfuron; PM-ch + aa 0 = potato multiplication media, no casein hydrolysate, amino acids, no chlorsulfuron; PM-ch + aa 10: potato multiplication media, no casein hydrolysate, amino acids, 10 μg/l chlorsulfuron; PM-ch + aa 20 = potato multiplication media, no casein hydrolysate, amino acids, 20 μg/l chlorsulfuron; amino acids refers to the presence of 100 mM each of leucine, isoleucine and valine. (DOCX 16 kb) [file 12896_2017_371_MOESM1_ESM.docx]

**Additional file 1: Figure S1.** Influence of culture medium composition and chlorsulfuron concentrations on *in vitro* plants of wild-type potato cultivar ‘Iwa’. Root length was measured after three weeks with mean root length (mm) ± standard deviation plotted (n=30). PM 0 = potato multiplication media, no chlorsulfuron; PM 10 = potato multiplication media, 10 μg/L chlorsulfuron; PM 20 = potato multiplication media, 20 μg/L chlorsulfuron; PM –ch 0 = potato multiplication media, no casein hydrolysate, no chlorsulfuron; PM –ch 10 = potato multiplication media, no casein hydrolysate, 10 μg/L chlorsulfuron; PM –ch 20 = potato multiplication media, no casein hydrolysate, 20 μg/L chlorsulfuron; PM-ch+aa 0 = potato multiplication media, no casein hydrolysate, amino acids, no chlorsulfuron; PM-ch+aa 10: potato multiplication media, no casein hydrolysate, amino acids, 10 μg/L chlorsulfuron; PM-ch+aa 20 = potato multiplication media, no casein hydrolysate, amino acids, 20 μg/L chlorsulfuron; amino acids refers to the presence of 100 mM each of leucine, isoleucine and valine.
