# Supplementary material for: Somatic cell selection for chlorsulfuron-resistant mutants in potato: identification of point mutations in the acetohydroxyacid synthase gene
Source: BMC Biotechnol. 2017 Jun 6;17:49. doi: 10.1186/s12896-017-0371-4 (PMC5461709; doi:10.1186/s12896-017-0371-4)
Supplement: Supplementary file 2 — A dose response experiment of chlorsulfuron on growth of wild-type potato cultivar ‘Iwa’ using PM medium without casein hydrolysate. Root length was measured after three weeks with mean root length (mm) ± standard deviation plotted (n = 30). (DOCX 15 kb) [file 12896_2017_371_MOESM2_ESM.docx]

**Additional file 2: Figure S2.** A dose response experiment of chlorsulfuron on growth of wild-type potato cultivar ‘Iwa’ using PM medium without casein hydrolysate. Root length was measured after three weeks with mean root length (mm) ± standard deviation plotted (n=30).
