# Supplementary material for: Somatic cell selection for chlorsulfuron-resistant mutants in potato: identification of point mutations in the acetohydroxyacid synthase gene
Source: BMC Biotechnol. 2017 Jun 6;17:49. doi: 10.1186/s12896-017-0371-4 (PMC5461709; doi:10.1186/s12896-017-0371-4)
Supplement: Supplementary file 3 — Alignment of the coding sequence of the wild-type potato cultivar ‘Iwa’ AHAS allele (GenBank accession HM114275) and partial DNA BAC sequences from the reference potato genome [35, 36]. Line 1: PGSC0003DMB000000227, which maps to chromosome 6; Line 2: PGSC0003DMB000000368, which maps to chromosome 3; Line 3: PGSC0003DMB000000096 which maps to chromosome 7; and Line 4: The coding region of the AHAS allele from ‘Iwa’, GenBank accession HM114275. (DOCX 1863 kb) [file 12896_2017_371_MOESM3_ESM.docx]

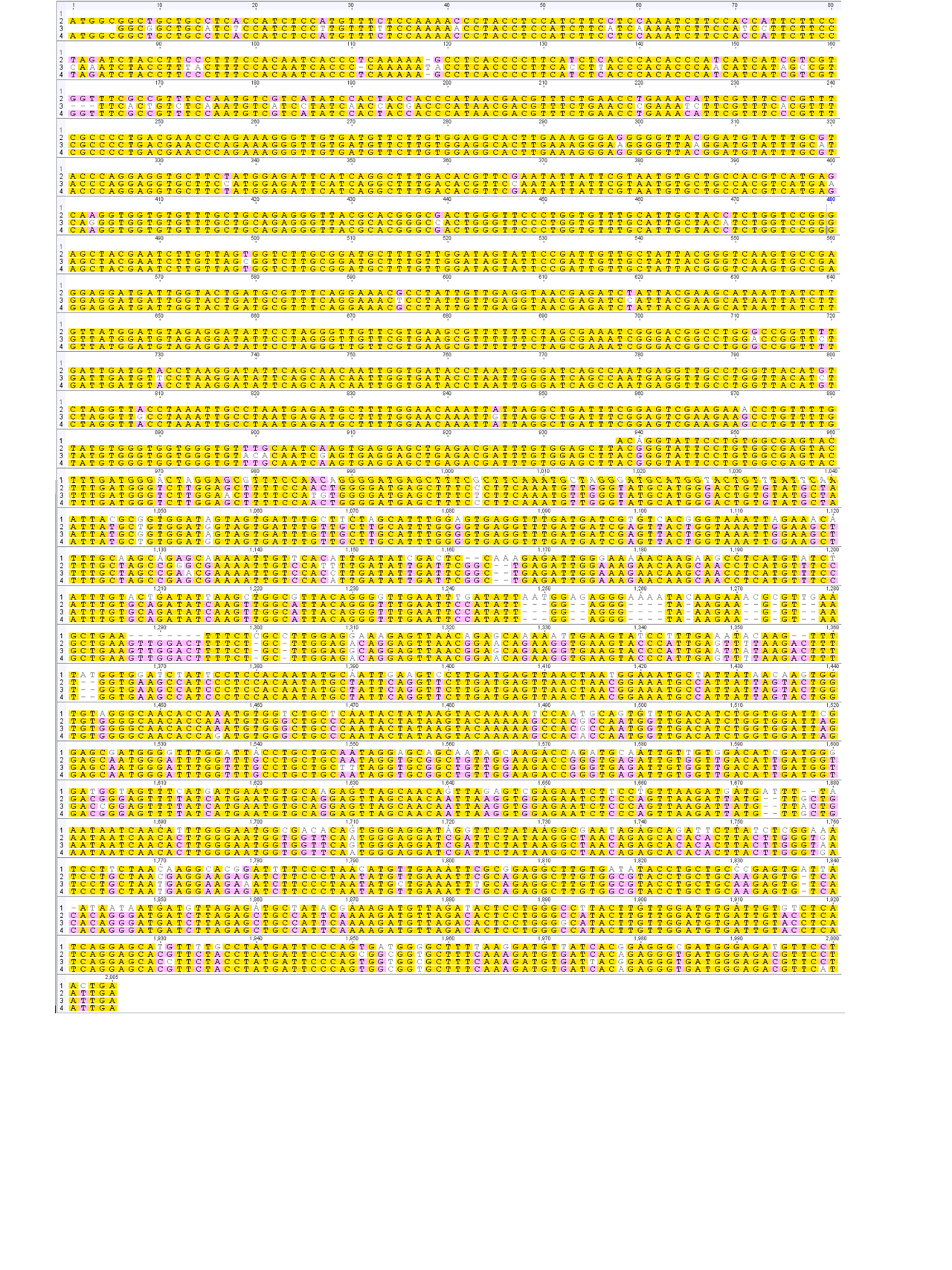


**Additional file 3: Figure S3**. Alignment of the coding sequence of the wild-type potato cultivar ‘Iwa’ *AHAS* allele (GenBank accession HM114275) and partial DNA BAC sequences from the reference potato genome [[35](#_ENREF_35), [36](#_ENREF_36)]. Line1: PGSC0003DMB000000227, which maps to chromosome 6; Line 2: PGSC0003DMB000000368, which maps to chromosome 3; Line 3: PGSC0003DMB000000096 which maps to chromosome 7; and Line 4: The coding region of the AHAS allele from ‘Iwa’, GenBank accession HM114275.
