# Supplementary material for: Somatic cell selection for chlorsulfuron-resistant mutants in potato: identification of point mutations in the acetohydroxyacid synthase gene
Source: BMC Biotechnol. 2017 Jun 6;17:49. doi: 10.1186/s12896-017-0371-4 (PMC5461709; doi:10.1186/s12896-017-0371-4)
Supplement: Supplementary file 4 — Nucleotide sequence of the mutated potato AHAS coding region. The two nucleotides highlighted in red are the point mutations identified in the somatic cell selection experiments from cell colonies CR06, CR27. The first mutation, the highlighted residue at nucleotide 556 was originally C and was converted to a T, which changes the 186th amino acid from a proline into a serine residue. The second highlighted residue was identified to have a mutation at position 1687, where a T residue was converted to A, which changed the 563rd amino acid from tryptophan into an arginine residue. (DOCX 12 kb) [file 12896_2017_371_MOESM4_ESM.docx]

ATGGCGGCTGCTGCCTCACCATCTCCATGTTTCTCCAAAACCCTACCTCCATCTTCCTCCAAATCTTCCACCATTCTTCC

TAGATCTACCTTCCCTTTCCACAATCACCCTCAAAAAGCCTCACCCCTTCATCTCACCCACACCCATCATCATCGTCGTG

GTTTCGCCGTTTCCAATGTCGTCATATCCACTACCACCCATAACGACGTTTCTGAACCTGAAACATTCGTTTCCCGTTTC

GCCCCTGACGAACCCAGAAAGGGTTGTGATGTTCTTGTGGAGGCACTTGAAAGGGAGGGGGTTACGGATGTATTTGCGTA

CCCAGGAGGTGCTTCTATGGAGATTCATCAGGCTTTGACACGTTCGAATATTATTCGTAATGTGCTGCCACGTCATGAGC

AAGGTGGTGTGTTTGCTGCAGAGGGTTACGCACGGGCGACTGGGTTCCCTGGTGTTTGCATTGCTACCTCTGGTCCGGGA

GCTACGAATCTTGTTAGTGGTCTTGCGGATGCTTTGTTGGATAGTATTCCGATTGTTGCTATTACGGGTCAAGTGTCGAG

GAGGATGATTGGTACTGATGCGTTTCAGGAAACGCCTATTGTTGAGGTAACGAGATCTATTACGAAGCATAATTATCTTG

TTATGGATGTAGAGGATATTCCTAGGGTTGTTCGTGAAGCGTTTTTTCTAGCGAAATCGGGACGGCCTGGGCCGGTTTTG

ATTGATGTACCTAAGGATATTCAGCAACAATTGGTGATACCTAATTGGGATCAGCCAATGAGGTTGCCTGGTTACATGTC

TAGGTTACCTAAATTGCCTAATGAGATGCTTTTGGAACAAATTATTAGGCTGATTTCGGAGTCGAAGAAGCCTGTTTTGT

ATGTGGGTGGTGGGTGTTTGCAATCAAGTGAGGAGCTGAGACGATTTGTGGAGCTTACGGGTATTCCTGTGGCGAGTACT

TTGATGGGTCTTGGAGCTTTTCCAACTGGGGATGAGCTTTCCCTTCAAATGTTGGGTATGCATGGGACTGTGTATGCTAA

TTATGCTGTGGATGGTAGTGATTTGTTGCTTGCATTTGGGGTGAGGTTTGATGATCGAGTTACTGGTAAATTGGAAGCTT

TTGCTAGCCGAGCGAAAATTGTCCACATTGATATTGATTCGGCTGAGATTGGAAAGAACAAGCAACCTCATGTTTCCATT

TGTGCAGATATCAAGTTGGCATTACAGGGTTTGAATTCCATATTGGAGGGTAAAGAAGGTAAGCTGAAGTTGGACTTTTC

TGCTTGGAGACAGGAGTTAACGGAACAGAAGGTGAAGTACCCATTGAGTTTTAAGACTTTTGGTGAAGCCATCCCTCCAC

AATATGCTATTCAGGTTCTTGATGAGTTAACTAACGGAAATGCCATTATTAGTACTGGTGTGGGGCAACACCAGATGTGG

GCTGCCCAATACTATAAGTACAAAAAGCCACACCAATGGTTGACATCTGGTGGATTAGGAGCAATGGGATTTGGTTTGCC

TGCTGCAATAGGTGCGGCTGTTGGAAGACCGGGTGAGATTGTGGTTGACATTGATGGTGACGGGAGTTTTATCATGAATG

TGCAGGAGTTAGCAACAATTAAGGTGGAGAATCTCCCAGTTAAGATTATGTTGCTGAATAATCAACACTTGGGAATGGTG

GTTCAAAGGGAGGATCGATTCTATAAGGCTAACAGAGCACACACTTACTTGGGTGATCCTGCTAATGAGGAAGAGATCTT

CCCTAATATGTTGAAATTCGCAGAGGCTTGTGGCGTACCTGCTGCAAGAGTGTCACACAGGGATGATCTTAGAGCTGCCA

TTCAAAAGATGTTAGACACTCCTGGGCCATACTTGTTGGATGTGATTGTACCTCATCAGGAGCACGTTCTACCTATGATT

CCCAGTGGCGGTGCTTTCAAAGATGTGATCACAGAGGGTGATGGGAGACGTTCATATTGA

**Additional file 4: Supplementary Figure S4**. Nucleotide sequence of the mutated potato *AHAS* coding region. The two nucleotides highlighted in red are the point mutations identified in the somatic cell selection experiments from cell colonies CR06, CR27. The first mutation, the highlighted residue at nucleotide 556 was originally C and was converted to a T, which changes the 186^th^ amino acid from a proline into a serine residue. The second highlighted residue was identified to have a mutation at position 1687, where a T residue was converted to A, which changed the 563^rd^ amino acid from tryptophan into an arginine residue.
