# Supplementary material for: Somatic cell selection for chlorsulfuron-resistant mutants in potato: identification of point mutations in the acetohydroxyacid synthase gene
Source: BMC Biotechnol. 2017 Jun 6;17:49. doi: 10.1186/s12896-017-0371-4 (PMC5461709; doi:10.1186/s12896-017-0371-4)
Supplement: Supplementary file 5 — The binary vector pMOA33-AHAS. The locations of the primers pMOA33RBFor and StCabAHASSeqRev used to confirm transgenic status of the regenerated plants are indicated. (DOCX 68 kb) [file 12896_2017_371_MOESM5_ESM.docx]

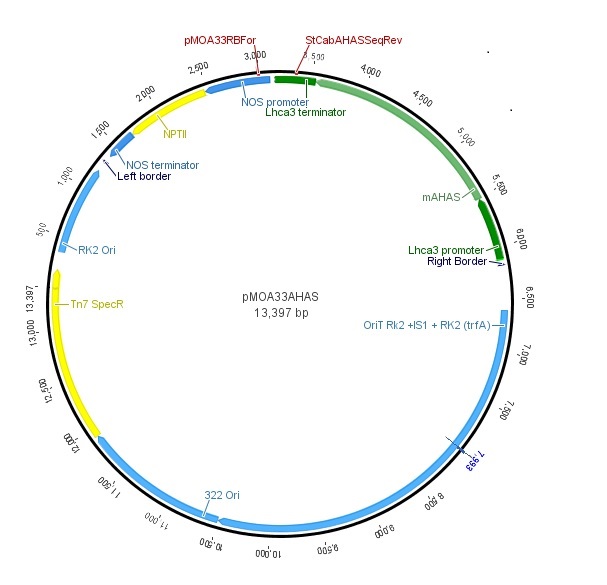


**Additional file 5: Figure S5.** The binary vector pMOA33-AHAS. The locations of the primers pMOA33RBFor and StCabAHASSeqRev used to confirm transgenic status of the regenerated plants are indicated.
